# Financial toxicity in lower urinary tract symptoms amongst men

**DOI:** 10.1186/s12894-025-01895-4

**Published:** 2025-08-21

**Authors:** Wilson Sui, Pablo Suarez, Heiko Yang, Maria Camila Velasquez Escobar, Feres Camargo Maluf, Taylor Hall, Sultan Al Azzawi, Lavanya Gupta, Thomas Chi

**Affiliations:** 1 Department of Urology, University of Michigan Medical School, 1500 E Medical Center Dr, Ann Arbor, MI 48109 USA; 2 Department of Urology, University of California San Francisco, San Francisco, CA USA; 3 Department of Urology, University of Colorado, Aurora, CO USA; 4 Department of Urology, University of Alabama at Birmingham, Birmingham, AL USA; 5 University of California Berkeley, Berkeley, CA USA

**Keywords:** Prostatic hyperplasia, Financial stress, Health expenditures, Lower urinary tract symptoms, Urinary incontinence

## Abstract

**Background:**

Financial toxicity (FT), first reported in oncologic patients and generally defined as harm to patients caused by the cost of treatment, is less well described in non-malignant urology. In chronic conditions related to lower urinary tract symptoms (LUTS), such as BPH, treatment costs may result in a significant substantial burden. The goal of this study was to characterize the association between FT and LUTS.

**Methods:**

A cross-sectional web-based survey was administered to a random sample of adult men through a national registry of volunteers (ResearchMatch). Disease-specific information, validated symptom scores, and an 11-item measure of LUTS-related financial toxicity were used to characterize participants. Multivariable logistic regression was performed to identify predictors of financial toxicity.

**Results:**

A total of 294 respondents with a self-reported history of BPH-associated LUTS were included, 41% of whom met the criteria for financial toxicity. Men with FT had worse LUTS symptom scores across all measures (*p* < 0.001). The presence of stress, urge, or mixed incontinence was significantly higher in men with FT (52% vs. 8%, 46% vs. 15%, and 36% vs. 5% respectively; *p* < 0.001). Men with FT spent more out-of-pocket on incontinence products than those without FT (*p* < 0.001). On multivariable analysis, mixed incontinence was a predictor of FT (OR 3.233, 95% CI 1.15–9.084).

**Conclusion:**

Two of five men with LUTS met the criteria for financial toxicity. These men had worse urinary symptom scores, higher rates of all types of incontinence, and higher out-of-pocket costs for incontinence products.

**Supplementary Information:**

The online version contains supplementary material available at 10.1186/s12894-025-01895-4.

## Introduction

Lower urinary tract symptoms (LUTS) are one of the most common presenting complaints among men seeking urologic care, which significantly reduces health status and quality of life [1–3]. The prevalence of the LUTS, most commonly attributed to BPH, increases with age with 44% of men in their 40 s and 50 s complaining of these symptoms and over 70% of men > 80 years old [4, 5]. Estimated annual costs for LUTS/BPH to the private sector approach $4 billion a year [6]. Given the high prevalence of BPH and the aging U.S. population, the financial burden of this disease is likely to continue to increase.

In an era of unsustainable healthcare expenditure in the United States, out-of-pocket costs for patients are also increasing as commercial insurers shift financial burden onto patients. Out-of-pocket prescription costs accounted for over $300 billion in 2010 which was 11.8% of total national health expenditures [7]. Medical management of male LUTS is the preferred first-line therapy with up to 60% of these men having filled prescription for LUTS/BPH [8, 9]. In addition, approximately one-fifth of all men with LUTS/BPH also have a diagnosis of OAB, and require medications or incontinence products for management of OAB symptoms related to detrusor overactivity. The direct costs related to OAB treatment and routine care items, such as incontinence products, over decades, have resulted in significant out-of-pocket costs for patients and a projected national cost of up to $82.6 billion [9, 10].

Financial toxicity is generally defined as harm to patients caused by the cost of treatment. Patients with financial toxicity are more likely to delay or forgo care completely which leads to poor outcomes [11]. Amongst cancer patients, the prevalence has been reported as high as 48% [12]. While substantial effort has been applied to studying financial toxicity in the oncologic population [13–15], there has been comparatively less work in the benign urologic population [16].

In efforts to understand financial toxicity in benign urology, a large medical survey collaborative was utilized to contact all male patients with a diagnosis of LUTS. The goal of this study was to evaluate the association between LUTS and financial toxicity using the validated questionnaire COmprehensive Score for financial Toxicity (COST) [17].

## Methods

### Recruitment and survey materials

Institutional review board approval was obtained (IRB #23-38991). An anonymous survey was created using RedCap (See Additional File 1) and then distributed using ResearchMatch™, a secure, national, online medical survey collaborative from September 2022 to February 2023 [18]. Participants were incentivized with a chance to be 1 of 10 participants to be randomly selected to receive $50. Of the 9,823 men contacted, 1,120 responded for a 11.4% response rate (Online Resource 1). Men with a history of stroke or paraplegia, incomplete questionnaires, or lack of self-reported LUTS were excluded, resulting in a final analytic cohort of 294 patients (See Additional File 2). Patients who reported any history of surgical treatment for BPH at any point in their lifetime were included in the study. A sensitivity analysis was conducted excluding the patients with history of BPH surgeries to determine its impact on FT associative factors. To assess urinary symptoms, we used three surveys: (1) the standard seven-item International Prostate Symptom Score (IPSS), (2) the International Consultation on Incontinence Questionnaire – Urinary Incontinence Short Form (ICIQ-UI SF), and (3) the Lower Urinary Tract dysfunction research network symptom index-10 (LURN SI-10) (Online Resource 2). IPSS scores are categorized into mildly symptomatic (0–7), moderately symptomatic (8–19),and severely symptomatic (20–35) [19]. The ICIQ-UI SF is scored out of 21 points, with higher points associated with greater symptom severity. Participants were categorized into “slight” (ICIQ-UI 1–5), “moderate” (ICIQ-UI SF 6–12), and “severe or very severe” (ICIQ-UI SF ≥ 13) [20, 21].

Urge urinary incontinence (UUI) and stress urinary incontinence (SUI) were identified using responses from the LURN SI-10 questionnaire as previously described [22]. UUI was defined as a positive response to the question “In the past 7 days, how often did you leak urine or wet a pad after feeling a sudden need to urinate?” SUI was defined as a positive response to the question “In the past 7 days, how often did you leak urine or wet a pad while laughing, sneezing, coughing, or doing physical activities such as exercising or lifting a heavy object?” Mixed urinary incontinence (MUI) was defined as those who had both SUI and UUI. Disposable incontinence product use was evaluated through questions about the utilization of liners, pads, or diapers for urinary leakage, using a dichotomous response. For those who indicated usage of any products, the number and cost were estimated by the participants based on their personal usage and expenditures. Outliers (>$500 per week) were excluded.

The COmprehensive Score for financial toxicity (COST) survey is a validated tool to assess financial distress in healthcare via self-reported outcomes [17]. This 11-item measure of healthcare-associated financial toxicity is scored from 0 to 44 with scores ≤ 25 representing moderate and severe financial burden [17, 23]. This validated survey, which has been previously evaluated in non-oncologic settings as well, was adapted to assess LUTS-associated FT [24].

### Statistical analysis

Descriptive statistics were performed to compare demographic and disease-specific characteristics and symptom scores between those with financial toxicity and those with lower financial burdens. Student T-tests and Pearson’s chi-squared test were performed for continuous and categorical respectively. Multivariable logistic regression was used to identify predictors of financial toxicity, adjusting for participant demographics, disease-specific factors, and urinary symptom scores.

## Results

A total of 294 respondents with a self-reported history of LUTS related to BPH were included, 41% of whom met criteria FT. Men with FT were younger, and more likely to be non-white or Hispanic (Table 1, all *p* < 0.001). In addition, they had lower rates of advanced degrees, higher employment rates, were more likely to be insured by Medicaid or Medicare, and to be single (Table 1, *p* < 0.05). Comorbidity differences included higher rates of type 2 diabetes in the FT group (42% vs. 33%, *p* = 0.002) (Table 1).

**Table 1:** Demographic and clinical characteristics of study cohort by financial burden

	Lower financial burden % (*n* = 173)	Financial Toxicity % (*n* = 121)	*p*-value
Age (years, mean ± SD)	67 14	45 16	< 0.001
Race			
White	95 (165)	58 (70)	
Non-white	5 (8)	42 (51)	< 0.001
Ethnicity			
Hispanic	96 (165)	84 (100)	
Non-Hispanic	4 (7)	16 (19)	< 0.001
Education			
College graduate	20 (34)	41 (49)	
High school or partial college	44 (75)	38 (45)	
Professional school	37 (63)	22 (26)	< 0.001
Employment			
Employed or current student	34 (58)	73 (88)	
Retired	65 (111)	18 (22)	
Out of work or unable to work	2 (3)	9 (11)	< 0.001
Relationship status			
Married/committed relationship	79 (134)	65 (78)	
Single	17 (29)	31 (38)	
Divorced	4 (7)	4 (5)	0.016
Insurance Status			
Private	27 (47)	35 (42)	
Medicare, VA or other insurance	70 (121)	46 (55)	
Medicaid, uninsured	3 (5)	20 (24)	< 0.001
Past Medical History			
Hypertension	46 (8)	38 (46)	0.161
Hyperlipidemia	30 (52)	22 (26)	0.101
Type 2 Diabetes	19 (33)	35 (42)	0.002
Obesity	17 (30)	17 (21)	0.997
COPD	5 (8)	7 (8)	0.46

Continuous variables expressed as mean +/- standard deviation and categorical variables as % (n). Statistical analysis performed includes 2-sample T-test or Pearson’s chi-square test respectively

*Abbreviations*: *COPD* Chronic Obstructive Pulmonary Disease

Men with FT were more likely to have had a prior BPH surgery (28% vs. 39%, *p* = 0.039) (Table 2). These men also reported greater use of any incontinence product use (24% vs. 35%, *p* = 0.035) (Table 2). The mean number of pads, diapers, and total incontinence products used per week was higher in men with financial toxicity, which in turn yielded higher mean out-of-pocket cost ($6 ± $41 vs. $33 ± $91, *p* < 0.001) (Table 2).

**Table 2:** Financial and treatment-related factors by financial burden

	Lower financial burden % (*n* = 173)	Financial Toxicity % (*n* = 121)	*p*-value
COST score	37 ± 5	19 ± 6	< 0.001
History of BPH surgery	28 (47)	39 (47)	0.039
History of acute urinary retention	3 (5)	5 (6)	0.358
History of UTI	5 (9)	6 (7)	0.828
Evaluated by urologist in last year	10 (18)	6 (7)	0.162
Incontinence product utilization			
Any prior incontinence use	24 (41)	35 (42)	0.035
Mean pads per week	0.2 ± 1	0.7 ± 1	< 0.001
Mean diapers per week	0.3 ± 1	0.7 ± 2	< 0.001
Mean total incontinence product per week	0.4 ± 2	1.4 ± 3	< 0.001
Mean self-reported cost per week ($)	6 ± 41	33 ± 91	< 0.001
Symptom scores			
ICIQ-UI			
Slight (≤ 5)	17 (29)	2 (3)	
Moderate (6–12)	51 (88)	49 (59)	
Severe or very severe (≥ 13)	32 (56)	49 (59)	< 0.001
IPSS score			
Mild (0–7)	59 (102)	17 (21)	
Moderate (8–19)	31 (54)	53 (64)	
Severe (≥ 20)	10 (17)	39 (36)	< 0.001
LURN SI-10			
<7	29 (49)	5 (6)	
≥ 7	72 (124)	95 (115)	< 0.001

Continuous variables expressed as mean +/- standard deviation and categorical variables as % (n). Statistical analysis performed includes 2-sample T-test or Pearson’s chi-square test respectively

*Abbreviations*: *COST* COmprehensive score for financial Toxicity, *BPH* Benign prostate hyperplasia, *UTI* Urinary tract infection, *IPSS* International Prostate Symptom Score, *ICIQ-UI* International Consultation on Incontinence Questionnaire – Urinary Incontinence), *LURN SI-10* Lower urinary tract dysfunction research network symptom index-10

**Fig. 1:**
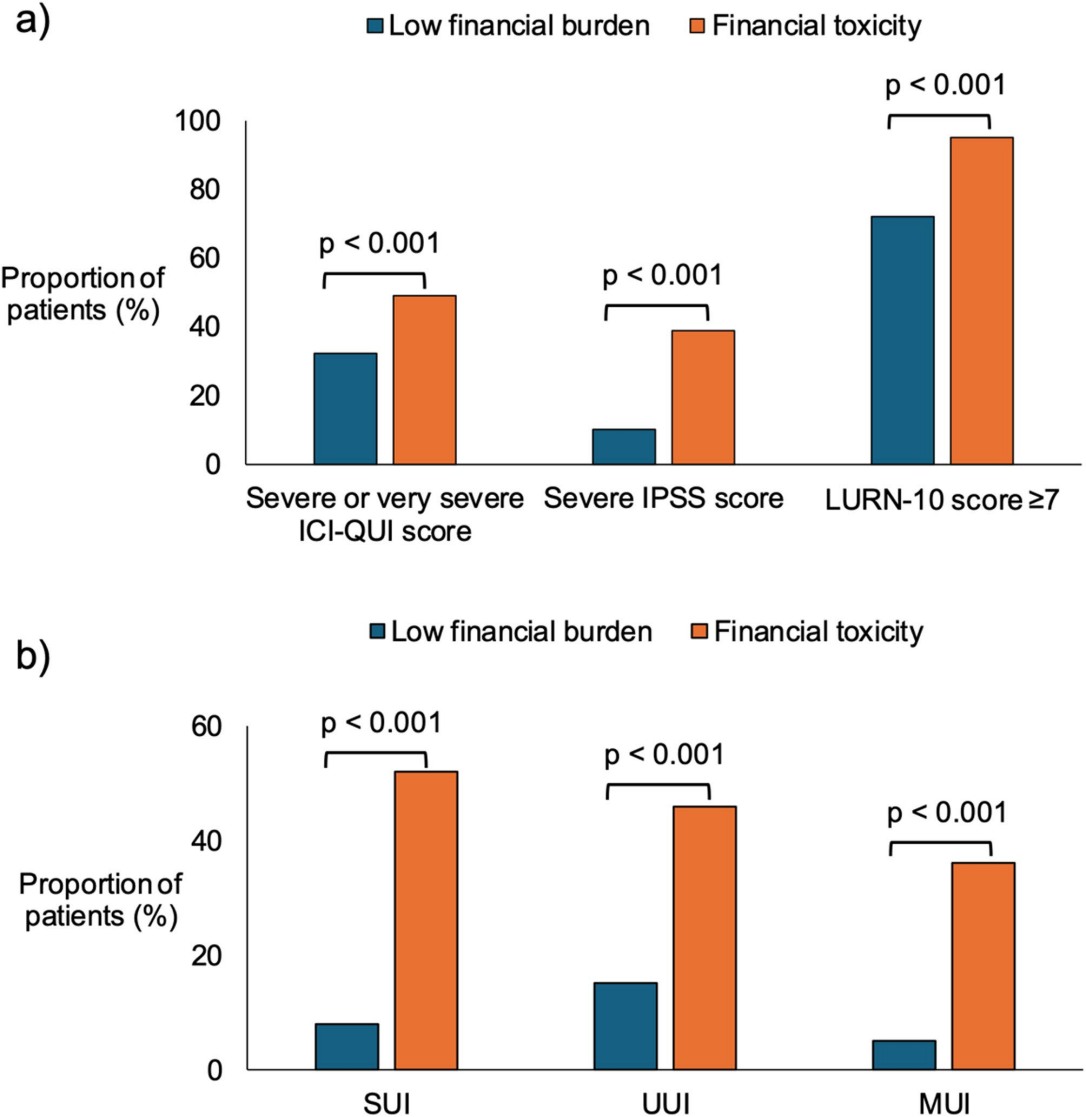
Association of LUTS symptom severity and urinary incontinence on financial toxicity. This figure compares financial toxicity (orange) versus low financial burden (blue) amongst patients with severe or very severe scores on ICI-QUI, IPSS, and LURN-10 assessments (**a**) and amongst those who met the criteria for stress (SUI), urge (UUI), and mixed urinary incontinence (MUI) (**b**)

On the assessment of symptom scores, men with FT were more likely to have severe or very severe ICI-QUI scores (32% vs. 49%, *p* < 0.001), severe IPSS scores (10% vs. 39%, *p* < 0.001) and LURN-10 SI scores ≥ 7 (72% vs. 95%, *p* < 0.001) (Fig. 1a). When compared to those with low financial burden, the FT group had higher rates of SUI (52% vs. 8% *p* < 0.001), UUI (46% vs. 15%, *p* < 0.001), and MUI (36% vs. 5%, *p* < 0.001) (Fig. 1b).

Age, race, and education were significant predictors of financial toxicity on multivariable logistic regression. Among urinary symptoms, IPSS scores were not associated with financial toxicity. However, mixed incontinence was linked to a significantly higher risk of financial toxicity (OR 3.23, 95% CI 1.15–9.08) (Table 3). A sensitivity analysis excluding patients with a history of BPH surgery (Supplementary Table 1) confirmed that age and race remained significant predictors, while the association with mixed LUTS was no longer observed.

**Table 3:** Multivariable logistic regression evaluating for predictors of financial toxicity

	OR (95% CI)	*p*-value
Age	0.941(0.914–0.969)	< 0.001
Race		
White	ref	ref
Non-white	4.305(1.567–11.827)	0.005
Education		
College graduate	ref	ref
High school or partial college	0.543(0.248–1.19)	0.127
Professional school	0.408(0.175–0.949)	0.037
Employment		
Employed or current student	ref	ref
Retired, out of work or unable to work	1.332(0.542–3.277)	0.532
Type 2 Diabetes	1.654(0.799–3.423)	0.175
Prior BPH surgeries	0.854(0.404–1.803)	0.678
OAB	0.861(0.379–1.955)	0.72
IPSS	1.041(0.992–1.092)	0.1
Type of incontinence		
None	ref	ref
UUI or SUI alone	1.279(0.504–3.244)	0.605
Mixed	3.233(1.15–9.084)	0.026

*Abbreviations*: *OR* Odds ratio, *CI* confidence Interval, *BPH* Benign prostate hyperplasia, *OAB* Overactive bladder, *IPSS* International Prostate Symptom Score, *UUI* Urge urinary incontinence, *SUI* Stress urinary incontinence

## Discussion

This study has 3 principal findings. One, 41% of men with self-reported LUTS related to BPH experience financial toxicity. Two, men with LUTS who experienced financial toxicity had worse symptom scores across all three urinary assessment tools and faced higher out-of-pocket expenses for incontinence products. Three, mixed incontinence was common amongst men with LUTS and was a significant predictor of financial toxicity after adjustment on multivariable regression.

Financial toxicity was first defined and validated in oncology due to the significant financial burden associated with medical treatment [13–15]. Patients with financial toxicity may delay or even forego care completely leading to sub-optimal outcomes [11]. There has been comparatively less effort in evaluating the role of financial toxicity in benign conditions [16, 25, 26]. These conditions are often chronic diseases, such as BPH/LUTS, which are common in the U.S. with over 40% of adults living with at least two [24]. Their economic burden is widely significant attributing to higher rates of unemployment, missed work, and being forced to assume fewer renumerating positions to accommodate their disease. Yet, the impact that the stress and financial burden this condition has on a person’s livelihood remain generally underrecognized in chronic illness.

In non-oncologic urology, LUTS related to BPH is a chronic disease with significant social and economic repercussions in a person’s life. An analysis of the Global Burden of Disease project demonstrated that the years lived with disability for BPH/LUTS was three times higher than that of prostate cancer, the 2nd highest source of years lived with disability, and growing at a faster rate than any other source of urologic-related morbidity [27]. In addition, the direct costs of LUTS treatments present an additional burden that along with disability further contributes to the impact of financial toxicity [28]. For instance, though many of the pharmacologic options for LUTS are generic, up to 60% of men facing these symptoms fill a related prescription [9]. These medications are then often utilized for decades resulting in substantial recurring out-of-pocket costs. In men with concomitant storage symptoms, the medication burden from anticholinergics or beta-3-agonists may be even more substantial. Furthermore, up to 30% of men do not achieve sufficient symptom control with medical management alone and require procedural intervention resulting in additional patient costs and loss of work [29].

To our knowledge, this is the first study to evaluate the association between financial toxicity and LUTS. Our results provide evidence that 40% men with LUTS experience financial toxicity. These individuals also demonstrated worse urinary symptom severity across all measures and higher rates of incontinence. Both stress and urge incontinence were higher in men with financial toxicity and therefore it is not surprising that these men also reported higher out-of-pocket costs for incontinence product use. While prior efforts have been undertaken to quantify female incontinence product usage and cost [22], this is the first report of product usage in male incontinence as that is often underrecognized with men unwilling to discuss their condition due to shame or embarrassment [30].

Men with FT had higher rates of all types of incontinence and mixed incontinence was found to be a predictor for FT after accounting for covariates. This finding may reflect greater symptom severity among respondents with FT, as evidenced by higher rates of prior BPH surgery in this group; additionally, those with persistent or postoperative incontinence may incur ongoing costs related to pharmacologic treatment and incontinence products, all of which contribute to a higher financial burden and increased risk of financial toxicity. Yet, the impact of BPH surgery on FT needs to be further studied. Although mixed incontinence was identified as a predictor of financial toxicity, this association did not persist in treatment-naïve men, as shown in the sensitivity analysis. We did not measure postoperative complications, treatment efficacy or appropriateness, may be important confounders. Nonetheless, this demonstrates the potential impact that surgical therapy may have on LUTS and FT, an important avenue for future research.

This study has several important limitations. First, the study survey was distributed anonymously over an electronic media therefore clarification or confirmation of reported responses was not feasible. Second, the generalizability of our study is limited by two main factors: 1) these findings reflect the experiences within the U.S. health care system, which differs significantly from countries with universal health coverage, where out-of-pocket expenses are minimal; 2) responses through ResearchMatch™, which draws from a nationwide economically diverse cohort, were skewed towards more highly educated population. To account for the differences between the study groups, particularly in age and ethnicity, we include potential confounders in our logistic regression. Yet, there remains the possibility of uncaptured confounders. For instance, we did not account for postoperative complications or the exact timing of surgery, which we believe represent an important area for future research to further refine patient stratification and outcomes. Third, we relied on patient recall of urinary symptoms, medical history, incontinence product use, and out-of-pocket costs which may have resulted in recall bias. Lastly, as with any survey-based study, it is also possible that individuals with more severe symptoms, particularly urinary incontinence, were more motivated to respond, potentially introducing selection bias in our sample. Selection bias may also result from the lack of preoperative data for patients who underwent BPH surgery at any point in their lifetime, limiting our ability to assess the true impact of surgical intervention on symptom burden. Patients who chose surgery likely had more severe baseline symptoms, which may have influenced the associations observed in this study.

The COST survey, although initially designed for oncology, has been broadly used in non-oncologic disease such as urinary incontinence and nephrolithiasis [22, 25, 26]. In our study, we used a more conservative approach that other studies therefore our findings are likely underestimating the true association between financial toxicity and LUTS. We recognize that LUTS is a broad category of disease, and we cannot differentiate the underlying diagnosis of LUTS from IPSS and self-reported data alone. For instance, the financial toxicity cohort may likely represent a more comorbid population, especially as there was a significantly higher proportion of diabetics. This may in part explain the significantly worse LUTS at a younger average age compared to the patients without financial toxicity. In the future, studies should implement diagnostic tools to establish a clinical diagnosis and truly assess the impact of LUTS-related conditions, such as BPH and OAB, on FT. However, we did use validated assessment tools to measure urinary symptoms and incontinence severity and captured important demographic and medical comorbidities for adjustment. Finally, this study was based on a sample of patients from the United States healthcare system which may not be reflective of the patient experience in other countries which have universal health care. In addition, we did not capture different types of incontinence products or out-of-pocket costs for surgical intervention or medications.

Nonetheless, this study included almost 300 participants with LUTS from across the nation and incorporated several validated surveys of financial toxicity and measures of symptom severity. The high rate of financial toxicity, in a highly prevalent disease that can impact men for decades, suggests that LUTS leads not only to a substantial financial burden for the healthcare system but also for individual patients, especially those with concomitant storage symptoms. As new medical therapies for LUTS-related chronic diseases emerge, they are likely to influence healthcare costs and financial burden on patients. For example, in its latest update to the BPH guidelines, the American Urological Association officially acknowledged more than a dozen procedural treatment options, ranging from minimally invasive surgical therapies to laser enucleation [31]. Thus, future research should evaluate not only these therapies’ effect on clinical symptoms and patient outcomes, but also their potential to alleviate, or exacerbate, financial distress.

## Conclusions

Two of five men with LUTS met the criteria for financial toxicity. These men had worse urinary symptom scores, higher rates of all types of incontinence, and higher out-of-pocket costs for incontinence products. Those with less education attainment, younger age, non-white race, and reported mixed incontinence are at greater risk for financial toxicity. These data highlight a need to better address the financial burden of chronic disease associated with these symptoms in patient counseling and management. Moreover, future work should aim to explore mechanisms contributing to underlying disparities of FT.

## Supplementary Information

Supplementary Material 1.

Supplementary Material 2.

Supplementary Material 3.

## Data Availability

The datasets analyzed during the current study are available from the corresponding author on reasonable request.

## Abbreviations

BPH: Benign Prostatic Hyperplasia
COST: Comprehensive score for financial Toxicity
FT: Financial Toxicity
ICIQ: UI–International Consultation on Incontinence Questionnaire–Urinary Incontinence Short Form
IPSS: International Prostate Symptom Score
LURN SI: 10–Lower Urinary Tract Dysfunction Research Network Symptom Index–10
LUTS: Lower Urinary Tract Symptoms
OAB: Overactive Bladder
