# Supplementary material for: Financial toxicity in lower urinary tract symptoms amongst men
Source: BMC Urol. 2025 Aug 21;25:213. doi: 10.1186/s12894-025-01895-4 (PMC12372289; doi:10.1186/s12894-025-01895-4)
Supplement: Supplementary file 1 — Supplementary Material 1. [file 12894_2025_1895_MOESM1_ESM.pdf]

Additional File 1. RedCap Questionnaires

Demographics And BPH Medical History

In this study, we aim to better understand the impact of benign prostatic hyperplasia (BPH) on one's quality of life and finances. Your responses are valuable, even if you have not been diagnosed with BPH.

Have you previously been told you have benign prostatic hyperplasia by your doctor OR had surgery for benign prostatic hyperplasia?	◯ Yes<br>◯ No
Have you visited the ED/urgent care in last 4 weeks due to your benign prostatic hyperplasia diagnosis?	◯ Yes<br>◯ No
Age	________________________
Ethnicity: Are you of Hispanic, Latino/a, or Spanish origin?	◯ Yes<br>◯ No<br>◯ Prefer not to answer
Race: What is your race? (Select all that apply)	☐ American Indian or Alaska Native<br>☐ Asian<br>☐ Black or African American<br>☐ Native Hawaiian or Pacific Islander<br>☐ White<br>☐ Other: (Please specify)<br>☐ Prefer not to answer
If you selected "other" for the previous question, specify your race:	________________________
What gender do you identify as?	◯ Male<br>◯ Female<br>◯ Gender non-conforming/Non-binary<br>◯ Gender fluid<br>◯ Other<br>◯ Prefer not to answer
Please specify if you selected "other" for the previous question	________________________
Please select your relationship status:	◯ Single<br>◯ Married<br>◯ In a relationship, living with partner<br>◯ In a relationship, not living with partner<br>◯ Divorced/Separated
Please select your current education level	◯ Did not graduate high school<br>◯ High school graduate<br>◯ Some college<br>◯ College graduate<br>◯ Professional school graduate

Please select your employment status	◯ Employed for wages<br>◯ Self-employed<br>◯ Out of work and looking for work<br>◯ Out of work but not currently looking for work<br>◯ A student<br>◯ Military<br>◯ Retired<br>◯ Unable to work
Please select your CURRENT type of health insurance	◯ Uninsured<br>◯ Private Insurance/HMO<br>◯ Medicare<br>◯ Medicaid<br>◯ VA<br>◯ Other
I have been diagnosed with the following conditions (select all that apply):	☐ Hypertension<br>☐ Hyperlipidemia<br>☐ Diabetes<br>☐ Obesity<br>☐ COPD<br>☐ Heart Attack<br>☐ Stroke<br>☐ Inflammatory Bowel Disease (ie. Crohn's Disease, Ulcerative Colitis)<br>☐ Gout<br>☐ Cystinuria<br>☐ Paraplegia
Have you had any surgeries for benign prostatic hyperplasia treatment? (please skip if you have not been diagnosed with benign prostatic hyperplasia)	◯ Yes<br>◯ No
How many surgeries have you had for benign prostatic hyperplasia treatment IN THE LAST YEAR (HOLEP/Laser, TURP, & etc.) ? (please skip if you have not been diagnosed with benign prostatic hyperplasia)	________________________________
How many surgeries have you had for benign prostatic hyperplasia treatment in your lifetime (HOLEP/Laser, TURP & etc.)? (please skip if you have not been diagnosed with benign prostatic hyperplasia)	________________________________
What benign prostatic hyperplasia surgeries have you had in the past? (select all that apply)	☐ UroLiFT<br>☐ ReZUM<br>☐ Transurethral resection of prostate (TURP)<br>☐ Holmium laser enucleation of prostate (HoLEP)<br>☐ Simple prostatectomy<br>☐ Other
If you selected "other" for the previous question, specify the surgery:	________________________________
In the past, have you not been able to urinate?	◯ Yes<br>◯ No
How many times have you not been able to urinate over the past year?	◯ Yes<br>◯ No

Have you ever had a Foley catheter inserted?	◯ Yes<br>◯ No
How many times have you had a Foley catheter inserted?	◯ Yes<br>◯ No
Have you ever been diagnosed with a urinary tract infection?	◯ Yes<br>◯ No
How many times have you been diagnosed with a urinary tract infection?	________________________________
Did you receive treatment for a urinary tract infection?	◯ Yes<br>◯ No
Do you have an urologist (A urologist is doctor who specializes in diagnosing and treating diseases of the urinary system)?	◯ Yes<br>◯ No
Does a urologist or other healthcare professional manage your benign prostatic hyperplasia diagnosis?	◯ A urologist manages my care<br>◯ Other healthcare professionals manage my care
How many appointments have you had with a urologist this year?	________________________________
If you have a urologist, how many appointments have you missed this year?	________________________________
At this moment, do you have a clean intermittent catheterization, indwelling urethral Foley catheter or suprapubic catheter inserted?	◯ NO I do not currently have a clean intermittent catheterization, indwelling urethral Foley catheter or suprapubic catheter inserted<br>◯ YES I have a clean intermittent catheterization<br>◯ YES I have a urethral Foley catheter inserted<br>◯ YES I have a suprapubic catheter inserted

LURN SI-10

This questionnaire asks you about different urinary symptoms. Please read each question carefully, and then circle the response that best describes your symptoms.

		Never	A few times	About half the time	Most of the time	Every time
1)	In the past 7 days, how often did you feel a sudden need to urinate?	○	○	○	○	○
2)	In the past 7 days, how often did you leak urine or wet a pad after feeling a sudden need to urinate?	○	○	○	○	○
3)	In the past 7 days, how often did you leak urine or wet a pad while laughing, sneezing, lifting heavy objects, exercising or coughing?	○	○	○	○	○
4)	In the past 7 days, how often did you leak urine or wet a pad when doing physical activities, such as exercising or lifting a heavy object?	○	○	○	○	○
5)	In the past 7 days, how often did you have pain or discomfort in your bladder while it was filling?	○	○	○	○	○
6)	In the past 7 days, how often did you have a delay before you started to urinate?	○	○	○	○	○
7)	In the past 7 days, how often was your urine flow slow or weak?	○	○	○	○	○
8)	In the past 7 days, how often did you dribble urine just after zipping your pants or pulling up your underwear?	○	○	○	○	○

		(3 or fewer times a day)	(4-7 times a day)	(8-10 times a day)	(11 or more times a day)
9)	In the past 7 days, during waking hours, how many times did you typically urinate?	○	○	○	○

		(none)	(1 time)	(2-3 times)	(More than 3 times)
10)	In the past 7 days, during a typical night, how many times did you wake up and urinate?	○	○	○	○

11) In the past 7 days, how bothered were you by urinary symptoms?

- ○ Not at all bothered
- ○ Somewhat bothered
- ○ Very bothered
- ○ Extremely bothered

12) Q 1-10 Sum ____________________

Iciqui-sf

We would be grateful if you could answer the following questions, thinking about how you have been, on average, over the PAST FOUR WEEKS.

1) How often do you leak urine? (Tick one box)

- ◯ never
- ◯ about once a week or less often
- ◯ two or three times a week
- ◯ about once a day
- ◯ several times a day
- ◯ all the time

2) We would like to know how much urine you think leaks. How much urine do you usually leak (whether you wear protection or not)? (Tick one box)

- ◯ none
- ◯ a small amount
- ◯ a moderate amount
- ◯ a large amount

	0 not at all	1	2	3	4	5	6	7	8	9	10 a great deal
3) Overall, how much does leaking urine interfere with your everyday life? Please ring a number between 0 (not at all) and 10 (a great deal)	◯	◯	◯	◯	◯	◯	◯	◯	◯	◯	◯

4) ICIQ score: sum scores 3+4+5

____

5) When does urine leak? (Please tick all that apply to you)

- ☐ never - urine does not leak
- ☐ leaks before you can get to the toilet
- ☐ leaks when you cough or sneeze
- ☐ leaks when you are asleep
- ☐ leaks when you are physically active/exercising
- ☐ leaks when you have finished urinating and are dressed
- ☐ leaks for no obvious reason
- ☐ leaks all the time

Ipss

1)	Over the past month, how often have you had a sensation of not emptying your bladder completely after you finished urinating?	◯ Not at all<br>◯ Less than 1 time in 5<br>◯ Less than half the time<br>◯ About half the time<br>◯ More than half the time<br>◯ Almost always
2)	Over the past month, how often have you had to urinate again less than 2 hours after you finished urinating?	◯ Not at all<br>◯ Less than 1 time in 5<br>◯ Less than half the time<br>◯ About half the time<br>◯ More than half the time<br>◯ Almost always
3)	Over the past month, how often have you found you stopped and started again several times when you urinated?	◯ Not at all<br>◯ Less than 1 time in 5<br>◯ Less than half the time<br>◯ About half the time<br>◯ More than half the time<br>◯ Almost always
4)	Over the past month, how often have you found it difficult to postpone urination?	◯ Not at all<br>◯ Less than 1 time in 5<br>◯ Less than half the time<br>◯ About half the time<br>◯ More than half the time<br>◯ Almost always
5)	Over the past month, how often have you had a weak urinary stream?	◯ Not at all<br>◯ Less than 1 time in 5<br>◯ Less than half the time<br>◯ About half the time<br>◯ More than half the time<br>◯ Almost always
6)	Over the past month, how often have you had to push or strain to begin urination?	◯ Not at all<br>◯ Less than 1 time in 5<br>◯ Less than half the time<br>◯ About half the time<br>◯ More than half the time<br>◯ Almost always
7)	Over the past month, how many times did you most typically get up to urinate from the time you went to bed at night until the time you got up in the morning?	◯ None<br>◯ 1 time<br>◯ 2 times<br>◯ 3 times<br>◯ 4 times<br>◯ 5 or more times
8)	Total criteria point count:	______________________________

Social Needs Screener

Social Determinants of Health Screener

1)	Are you worried about anything in your living environment that may cause you to get sick? (has mold, bugs/rodents, water leaks, not enough heat etc.)	◯ Yes<br>◯ No
2)	Are you worried that in the next 2 months, you may not have a safe or stable place to live? (eviction, being kicked out, homelessness)	◯ Yes<br>◯ No
3)	In the last 12 months, did you worry that your food could run out before you got money to buy more?	◯ Yes<br>◯ No
4)	In the past 12 months, have you had challenges with paying for your medications?	◯ Yes<br>◯ No
5)	In the last 12 months, has lack of transportation kept you from medical appointments or getting your medications?	◯ Yes<br>◯ No
6)	In the last 12 months, did you have to skip buying medications or going to doctor's appointments to save money?	◯ Yes<br>◯ No
7)	In the past 12 months, has the electric, gas, oil or water company threatened to shut off services to your home?	◯ Yes<br>◯ No
8)	How many people live in your household?	◯ 1<br>◯ 2<br>◯ 3<br>◯ 4 or more
9)	Are you finding it hard to get along with a partner, spouse, or family members?	◯ Yes<br>◯ No
10)	Do you need help getting child care or care for an elderly or sick adult?	◯ Yes<br>◯ No
11)	Do you need legal help? (child/family services, immigration, housing discrimination, domestic issues, etc.)?	◯ Yes<br>◯ No
12)	Does anyone in your life hurt you, threaten you, frighten you or make you feel unsafe?	◯ Yes<br>◯ No
13)	What is your households yearly income?	◯ $10,000-$24,999<br>◯ $25,000-$49,999<br>◯ $50,000-$74,999<br>◯ $75,000-$99,999<br>◯ $100,000-$149,999<br>◯ $150,000+

COST11

Below is a list of statements about finances AS IT RELATES TO YOUR PROSTATE DISEASE. Please select your response for each statement as it applies to the last 7 days.

		Not at all	A little bit	Somewhat	Quite a bit	Very much
1)	I know that I have enough money in savings, retirement, or assets to cover the cost of my benign prostatic hyperplasia treatment	○	○	○	○	○
2)	My out-of-pocket medical expenses are more than I thought they would be	○	○	○	○	○
3)	I worry about the financial problems I will have in the future as a result of my benign prostatic hyperplasia diagnosis or treatment	○	○	○	○	○
4)	I feel I have no choice about the amount of money I spend on care	○	○	○	○	○
5)	I am frustrated that I cannot work or contribute as much as I usually do	○	○	○	○	○
6)	I am satisfied with my current financial situation	○	○	○	○	○
7)	I am able to meet my monthly expenses	○	○	○	○	○
8)	I feel financial stressed	○	○	○	○	○
9)	I am concerned about keeping my job and income, including work at home	○	○	○	○	○
10)	My benign prostatic hyperplasia or treatment has reduced my satisfaction with my present financial situation	○	○	○	○	○
11)	I feel in control of my financial situation	○	○	○	○	○
12)	My benign prostatic hyperplasia diagnosis has been a financial hardship to my family and me	○	○	○	○	○

13) Financial Toxicity Scale

____

Incontinence Products

Have you ever worn disposable incontinence products including liners, pads, or diapers for urinary leakage?	◯ Yes<br>◯ No
How many pads have you used in the last 24 hours? (Type 0 if none)	______________________
How many diapers have you used in the last 24 hours? (Type 0 if none)	______________________
What is the total cost in dollars spent on incontinence products per week?	______________________
Total number of incontinence products used:	______________________
