# Supplementary material for: Financial toxicity in lower urinary tract symptoms amongst men
Source: BMC Urol. 2025 Aug 21;25:213. doi: 10.1186/s12894-025-01895-4 (PMC12372289; doi:10.1186/s12894-025-01895-4)
Supplement: Supplementary file 2 — Supplementary Material 2. [file 12894_2025_1895_MOESM2_ESM.pdf]

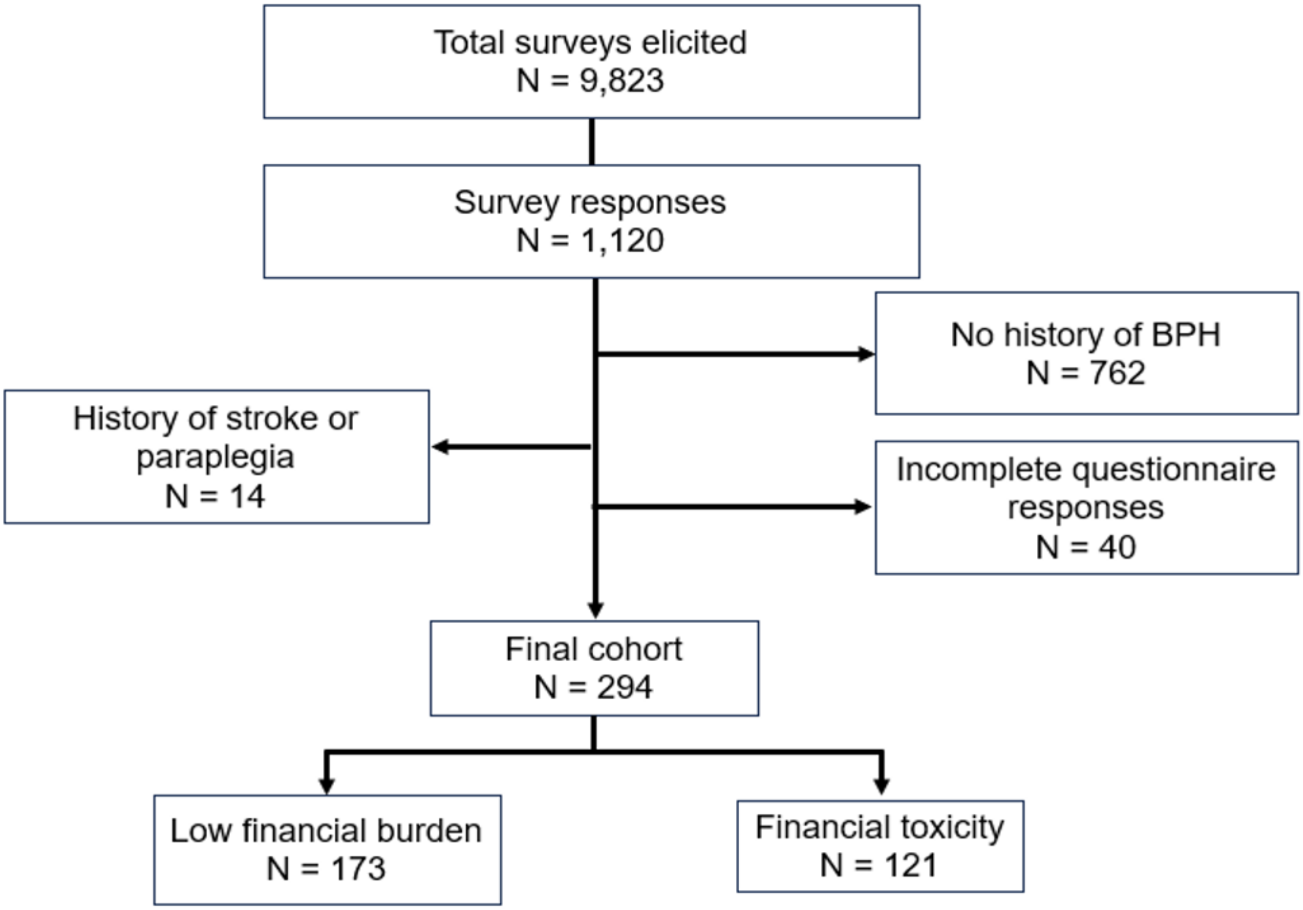

Additional File 2. Final Analytic Cohort

From the 9,823 surveys, the response rate was 3.1% or 1,120 responses. Exclusion criteria include no history of LUTS, history of stroke or paraplegia, and incomplete questionnaire responses. A total of 294 respondents met the study criteria, of whom 173 were categorized as having low financial burden and 121 as having financial toxicity.
