# Supplementary material for: Financial toxicity in lower urinary tract symptoms amongst men
Source: BMC Urol. 2025 Aug 21;25:213. doi: 10.1186/s12894-025-01895-4 (PMC12372289; doi:10.1186/s12894-025-01895-4)
Supplement: Supplementary file 3 — Supplementary Material 3. [file 12894_2025_1895_MOESM3_ESM.docx]

Additional File 3: Sensitivity Analysis

| Supplementary Table 1. Sensitivity analysis of predictors of financial toxicity | | |
| --- | --- | --- |
|  | OR (95% CI) | p-value |
| Age | 0.946(0.914 - 0.98) | 0.002 |
| Race |  |  |
| White | ref | ref |
| Non-white | 4.199(1.215 - 14.507) | 0.023 |
| Education |  |  |
| College graduate | ref | ref |
| High school or partial college | 0.552(0.219 - 1.39) | 0.207 |
| Professional school | 0.568(0.213 - 1.517) | 0.259 |
| Employment |  |  |
| Employed or current student | ref | ref |
| Retired, out of work or unable to work | 1.143(0.417 - 3.13) | 0.795 |
| Type 2 Diabetes | 1.561(0.64 - 3.81) | 0.327 |
| Prior BPH surgeries | - | - |
| OAB | 1.419(0.525 - 3.839) | 0.49 |
| IPSS | 1.028(0.969 - 1.09) | 0.36 |
| Type of incontinence |  |  |
| None | ref | ref |
| UUI or SUI alone | 1.859(0.58 - 5.962) | 0.297 |
| Mixed | 2.157(0.593 - 7.855) | 0.244 |
